# Factors Underlying Unmet Medical Needs: A Cross-Sectional Study

**DOI:** 10.3390/ijerph16132391

**Published:** 2019-07-05

**Authors:** Young Suk Yoon, Boyoung Jung, Dongsu Kim, In-Hyuk Ha

**Affiliations:** 1 Department of Korean Medicine Rehabilitation, Jaseng Hospital of Korean Medicine, 536 Gangnam-daero, Gangnam-gu, Seoul 06110, Korea; 2 Jaseng Spine and Joint Research Institute, Jaseng Medical Foundation, 3F, 538 Gangnam-daero, Gangnam-gu, Seoul 06110, Korea; 3 KM Policy Research Center, Korea Institute of Oriental Medicine, 1672, Yuseong-daero, Yuseong-gu, Daejeon 34054, Korea

**Keywords:** unmet medical need, health care system, Korea Health Panel Survey (KHPS), Anderson’s Behavioral Model

## Abstract

Unmet medical needs refer to the state where a patient’s medical care or service is insufficient, inadequate, or lacking. Numerous factors influence unmet medical needs. We used a multi-pronged approach to explore the factors influencing unmet medical needs in the Korean health care system according to Anderson’s Behavioral Model of Health Services Use. To this end, we used data from 11,378 adults over 19 years old in the 2016 Korea Health Panel Survey and performed multiple logistic regression analyses. The odds of experiencing unmet medical needs were significantly greater among older participants (odds ratio (OR) = 2.51, 95% confidence interval (CI) = 1.78–3.56); low-income participants (OR = 1.41, 95% CI = 1.14–1.75); non-workers (OR = 1.24, 95% CI = 1.06–1.46); those who had received non-covered treatment (OR = 1.24, 95% CI = 1.08–1.42); those who did not regularly exercise (OR = 1.23, 95% CI = 1.02–1.48); and those experiencing pain (OR = 2.29, 95% CI = 1.97–2.66), worse self-rated health status (OR = 2.29, 95% CI = 1.89–2.79), and severe depression (OR = 2.46, 95% CI = 1.39–4.35). About one in ten Korean citizens (11.60%) have unmet medical service needs. Policies that strengthen coverage for physically and economically vulnerable groups are needed.

## 1. Introduction

Health care should be of a certain quality and delivered in an acceptable and cost-effective manner to everyone in a society [1]. Naturally, however, people have varied health care demands, which can broadly be classified into medical needs and medical wants, depending on the decision maker [2]. Needs are based on expert judgments, while wants originate in the patients themselves. There is no universally accepted definition of ‘unmet need’, which is used differently by other commentators [3]. Reeves et al. (2015) defined unmet need as being unable to obtain care when people believed it to be medically necessary [4]. 

Among the 27 member countries of the Organization for Economic Co-Operation and Development (OECD), about 2.5% of patients have reported unmet medical needs [5]. The reasons provided for these unmet medical needs varied, and included financial reasons (Greece, Italy, Poland, and Portugal), wait time (Poland, Finland, and Estonia), and difficulty in transportation (Norway). The United Kingdom showed particularly low inequality between the upper and lower 50% of the population in terms of income level, whereas the United States exhibited the largest inequality between income levels, with 1/3 of adults among the lower 50% of the population in terms of income level experiencing unmet medical needs due to financial reasons [6]. According to the World Health Report, published by the World Health Organization (WHO), guaranteeing accessibility to medical care based on need rather than ability to pay is one method of improving population health [7]. Therefore, health care systems should be evaluated to ensure that individuals are receiving the medical services they need; one such evaluation measure is “the proportion of the population that has unmet needs.”

The Republic of Korea’s universal health care system has contributed to a marked improvement in population health conditions while also achieving one of the lowest levels of spending among OECD member countries through its implementation of high patient co-pays and limited public health insurance coverage [8]. The national health insurance (NHI) system designates “covered” and “non-covered” services. The latter are services that are not guaranteed for coverage by the NHI, which means that the patient must pay 100% of the treatment costs [9]. The coverage rate of the NHI (i.e., the number of covered services out of all services) in the past 10 years has been around 63.2%; this rate has become a point of criticism for many experts, who believe that the coverage is insufficient to offset the ever-increasing burden of medical costs for citizens [10]. Moreover, according to previous studies, the rate of unmet medical needs is higher than 10% [11,12]. Indicators of self-reported unmet medical needs must be evaluated alongside other indicators such as treatment possibility and accessibility, including the extent of health care coverage in that country, the amount of out-of-pocket payments, and actual use of health services [5]. 

Numerous influencing factors of unmet medical needs have been identified, and they can be broadly categorized as follows [13]: (1) availability, including long waiting times or unavailable services; (2) accessibility, including financial or transportation barriers; and (3) acceptability, including busyness or ignoring health problems [14]. Past studies on these factors have had different focuses, such as on women [11,15], income and health condition factors [16], the relationship of unmet medical needs with chronic diseases [17], household characteristics [18], costs [19], employment status [20], and habitation factors such as living on an island [21]. However, the factors influencing unmet medical needs will differ according to the characteristics of the health care system; past studies have typically only examined the relevance of individual patient characteristics, without consideration of the factors inherent in the larger health care system. To address this gap in the literature, we analyzed not only the individual characteristics related to unmet medical needs but also the characteristics of the Korean health care system (e.g., type of medical care, whether treatment is non-covered). The larger aim of this study is to devise policy suggestions on a country level, for example for Japan, France, Germany, and the Netherlands, with health care systems that are similar to that of the Republic of Korea. 

## 2. Materials and Methods 

### 2.1. Study Design and Population

We used data from the Korea Health Panel Survey (KHPS), a nationwide study conducted by a consortium formed by the Korea Institute of Health and Social Affairs and the National Health Insurance Corporation (KNHC). The purpose of the KHPS is to obtain basic data regarding health care utilization, health expenditures, and health-related behaviors, to be used in establishing policies related to achieving healthy living while limiting medical costs [22]. The KHPS has been conducted annually since 2008. The main goals of the development and dissemination of the KHPS are to provide the most versatile database possible on healthcare behaviors. Sample weights for the KHPS were calculated after adjusting for unequal selection probabilities and non-responses and making a population distribution disclosure via post-stratification corresponding to the sample distribution [23]. We used the 2016 yearly combined KHPS healthcare data collected in 2016 (beta version 1.5.1) for this cross-sectional study. The 2016 data is based on 6437 households and a total of 18,576 household members.

**Figure 1:**
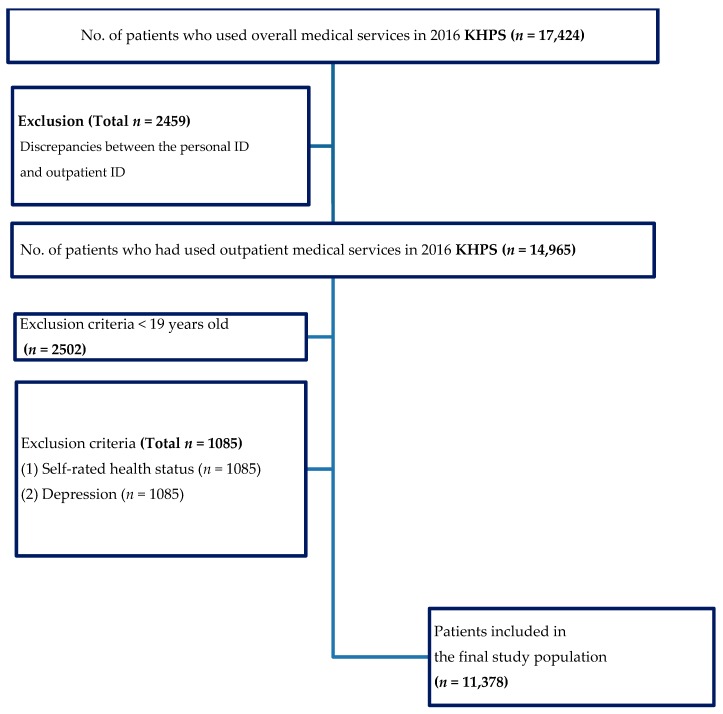
Flowchart of the study population. Abbreviations: KHPS = Korea Health Panel Survey.

For this study, we excluded respondents with discrepancies between the personal ID and outpatient ID. Additionally, individuals younger than 19 years of age were excluded, as were those people who provided no answers or erroneous responses to some items according to the KHP codebook. Also, we have had several expert meetings regarding operationalize the variable (income and type of medical care). Income was not equally distributed and the missing value is 1/4 of the raw data. Among people with a low income, there is a tendency in Korea to not respond to such items. Therefore, we did not remove missing data and analyzed them as belonging to a low-income group. The sample size of individuals who had received traditional Korean medicine was too small, so we had to operationalize the variable to people who had experienced traditional Korean medicine at least once in their lives. Finally, there were a total of 11,378 respondents for the final analyses and Figure 1 shows the participant flow.

### 2.2. Variables

The dependent variable of this study was whether respondents had experienced unmet medical needs. This was evaluated with a single item: “In the past year, have you ever needed hospital care or an examination but did not receive it?” Respondents who answered, “Yes, I have experienced a situation where I did not receive care at least once” were defined as having experienced unmet medical needs.

To analyze the factors that influence unmet medical needs, we followed Anderson’s Behavioral Model of Health Services Use [24,25,26]. The factors suggested in Anderson’s model can be categorized into three types:

(1) Predisposing factors: These characteristics are those that people possess, often regardless of their will, before the occurrence of a medical need. They include sex, age, education level, and marital status. Age was categorized into 10-year intervals, while education level was categorized into middle/high school graduation, college or university graduation, and graduate school graduation. For marital status, individuals who were married (including common-law marriage) were defined as married, and all other statuses were categorized as unmarried. 

(2) Enabling factors: These factors refer to the individual and community resources that enable the use of medical care. In this study, we focused on economic activity, total household income, medical insurance type, private insurance, and use of non-covered treatment costs. Economic activity, private insurance, and non-covered treatment costs were categorized as either “yes” or “no.” Household income was categorized into quintiles by income level. Medical insurance was categorized into three groups (NHI, Medicaid, or others).

(3) Need factors: These factors refer to those related to the level of disability or disease of an individual that are direct causes of the use of medical services. We considered the presence of certain chronic diseases, disability, regular exercise, and pain; self-rated health status; and level of depression. Regular exercise and pain were evaluated dichotomously (“yes/no”), while self-rated health status was categorized into good, moderate, and poor. Depression was categorized according to severity: light, moderate, and severe. We evaluated the presence of any of the following chronic diseases: high blood pressure, diabetes, hyperlipidemia, joint disease, tuberculosis, ischemic heart disease, and cerebrovascular disease.

### 2.3. Statistical Analysis

Categorical variables were presented as frequencies and percentages, whereas continuous variables (e.g., age) were presented as means and standard deviations. We converted costs into 2019 U.S. dollars (1 USD = 1133.5 KRW as of 5 April 2019). To examine the current status of unmet medical needs according to respondents’ characteristics, we calculated the frequencies and percentages of such needs and used the chi-square test for comparison.

Next, we performed multiple logistic regression analyses to identify the factors that influence unmet medical needs. We also conducted a stratified logistic regression analysis of the factors by age group (19–39, 40–59, 60 or older) and examined if the association of non-covered treatment costs differed by age, sex, and treatment type. The fit of the models was tested using the likelihood ratio and the area under the receiver operating characteristic curve (AUC). All analyses were performed using SAS 9.4 (SAS Institute, Cary, NC, USA) and IBM SPSS Statistics 25 (SPSS Inc., Chicago, IL, USA). 

### 2.4. Ethics Statement

Because of the retrospective nature of this study, which utilized data with encrypted personal information, it was granted an exemption from ethical approval in writing by the Institutional Review Board of Jaseng Hospital of Korean Medicine in Seoul, Korea (JASENG 2019-03-003). All authors read and followed the tenets of the Declaration of Helsinki in preparing this study.

## 3. Results

Table 1 shows the presence of unmet medical needs according to respondents’ characteristics. Out of the 11,378 total subjects, 1320 (11.60%) had experienced unmet medical needs. 

Table 2 shows the results of a similar analysis while also stratifying by age group. The following factors were associated with unmet medical needs in all age groups: among the predisposing factors, being female (*p* = 0.000); among the enabling factors, total household income (*p* = 0.000); and among the need factors, pain, self-related health status, and depression (*p* = 0.000).

Table 3 shows the results of the overall multiple logistic regression analysis. As can be seen in Model 1, the odds of unmet medical need were higher among the older age group (70–79 years old, odds ratio (OR) = 2.51, 95% confidence interval (CI) = 1.78–3.56), participants with a lower level of education (OR = 1.51, 95% CI = 1.24–1.85), and participants who were unmarried (OR = 1.63, 95% CI = 1.42–1.86). Model 2 revealed several more related factors, including income, type of health insurance, and private insurance, in addition to the Model 1 factors of being female and unmarried. The odds of unmet medical needs were higher among participants with a lower monthly income (OR = 1.49, 95% CI = 1.21–1.84), those receiving Medicaid (OR = 1.43, 95% CI = 1.13–1.82), and those without private insurance (OR = 1.17, 95% CI = 1.01–1.36). Model 3 revealed that the odds of unmet medical needs were significantly greater among participants who lacked employment (OR = 1.24, 95% CI = 1.06–1.46), those who were Medicaid recipients (OR = 1.48, 95% CI = 1.22–1.78), those who had received non-covered treatment (OR = 1.24, 95% CI = 1.08–1.42), those not regularly exercising (OR = 1.23, 95% CI = 1.02–1.48), those experiencing pain (OR = 2.29, 95% CI = 1.97–2.66), those who reported a bed self-rated health status (OR = 2.29, 95% CI = 1.89–2.79), and those who had severe depression (OR = 2.46, 95% CI = 1.39–4.35).

Table 4 shows the results of the regression analysis according to age group (19–39 years, 40–59 years, ≥60 years). The results indicated that, across all age groups, self-rated health status and depression were significant factors. For self-rated health status, the odds ratios of unmet medical needs were as follows: 19–39 years old (OR = 2.43, 95% CI = 1.32–4.48), 40–59 years old (OR = 2.23, 95% CI = 1.57–3.16), and ≥60 years old (OR = 2.34, 95% CI = 1.77–3.08). For depression, the odds ratios were as follows: 19–39 years old (OR = 2.26, 95% CI = 1.31–3.91), 40–59 years old (OR = 1.43, 95% CI = 1.08–1.89), and ≥60 years old (OR = 1.97, 95% CI = 1.63–2.39). For participants aged 19–39 years, the enabling factors (i.e., economic activity and income) had the largest association on unmet medical needs. Compared to participants in the highest income quintile (5Q), the odds of unmet medical needs increased as income decreased (2Q: OR = 2.32, 95% CI = 1.14–4.71; 3Q: OR = 3.83, 95% CI = 2.12–6.91). Compared to participants who were not participating in economic activities, those who did participate had higher odds of unmet medical needs, although economic activity did not have a large impact on the 40–59 years age group compared to the other age groups. The odds of unmet medical needs was higher among individuals with disability in the 40–59 years old group (OR = 1.62, 95% CI = 1.04–2.55), those who did not engage in regular exercise (OR = 1.68, 95% CI = 1.02–2.77), and those who experienced pain (OR = 2.82, 95% CI = 2.22–3.59), all of which hinder physical accessibility. Among participants who are ≥60 years, non-covered treatment increased the odds of unmet medical needs to a greater extent than in the other age groups (OR = 1.31, 95% CI = 1.08–1.58). Among the younger participants, marital status did not have an impact on unmet medical needs; however, it had a large association on the odds of unmet medical needs for middle-aged and older individuals (40–59: OR = 1.41, 95% CI = 1.06–1.87, 60 years≦: OR = 1.59, 95% CI = 1.31–1.92). 

Table 5 shows the association of non-covered treatment on the odds of unmet medical needs according to sex, age, and type of medical care. This factor was associated with unmet medical needs only in males (Model 3 OR = 1.47, 95% CI = 1.19–1.82), those ≥60 years old (Model 3 OR = 1.31, 95% CI = 1.08–1.58), and those who had only experience with Western medicine (Model 3 OR = 1.33, 95% CI = 1.15–1.55). 

Table A1 shows the mean costs of individuals with met medical needs and those with unmet medical needs according to type of medical care. When looking at the costs used in Western medicine and traditional Korean medicine, we found that the total treatment costs, health insurance costs, out-of-pocket costs, and non-covered treatment costs were higher among those who had experienced unmet medical needs in both types. The non-covered treatment costs of Western medicine were higher than were those of traditional Korean medicine (Western medicine: $111.39; traditional Korean medicine: $3.31). 

## 4. Discussion

This study examined the current status and correlates of unmet medical needs among Korean adults using data from the KHPS. The results indicate that 11.6% of Korean adults had experienced unmet medical needs, which is slightly lower than the 14.5% found in a previous study [27]. However, it is about five times as high as the 2.5% found among the 27 OECD countries in 2016 [5]. The largest reason for unmet medical needs was financial barriers (28.8%), which is identical to the findings of a previous study [20,28] (see Table A2). The correlates of unmet medical needs can be summarized as follows.

First, the predisposing factors were age, education level, and marital status. As shown in Table A3, the experience of unmet medical needs increased with age, which accords with the findings of previous research [29,30,31]. The rate was particularly high among those in their forties, possibly because this group has less time to access medical services due to their relatively active social lives [19]. The analyses by age offer some support for this, as the need factors that hindered physical accessibility, such as disability and pain, had a larger impact on this phenomenon than did financial reasons among individuals in their forties compared to other age groups (Table 4). As for education level, past research has found it to be associated with health behaviors and attitudes [11,32]. It is possible that the experience of unmet medical needs was more common among participants with lower education levels and those who were unmarried because they put less focus on health management [15,33]. 

Second, the enabling factors related to unmet medical needs were economic activity, monthly income, and medical insurance type. These associations, particularly the fact that the odds of unmet medical needs were higher for non-workers, those with lower monthly household incomes, those on Medicaid, and those with non-covered treatment costs, can be largely attributed to financial reasons [34]. The financial burden [5,35] experienced by unemployed persons and those from lower income households [36] can lead to unmet medical needs. The main cause of unmet medical needs was found to be financial reasons in other countries as well, including Greece, Italy, Poland, and Portugal [37,38]. In South Korea, Medicaid recipients are low-income individuals who are eligible for coverage when they require medical services due to disease, injury, or pregnancy [39]. The fact that Medicaid recipients experience unmet medical needs suggests that the NHI does not provide financially sufficient coverage. 

Non-covered treatments are defined as treatments for which persons must pay the costs along with their co-pays due to a limited scope of coverage [30]. According to the OECD Health Statistics 2015, 37% of the total health expenditures in South Korea was from out-of-pocket payments [40]. The proportion of households that incur so-called catastrophic medical costs—Being overburdened by non-covered medical expenses, which often culminates in bankruptcy—Increased from 3.68% in 2010 to 4.39% in 2013. The increase was to 12.86% among the lowest-income households [41]. Patients who receive non-covered Patients who receive non-covered treatment often have difficulty meeting their medical needs because of the burden of medical costs treatment often have difficulty meeting their medical needs because of the burden of medical costs [42]. The total medical costs, health insurance costs, co-pays, and non-covered cost amount were found to be higher among individuals who had experienced unmet medical needs (Table A1). Therefore, policies aimed at reducing the financial burdens of patients, such as by reducing the number of non-covered treatments through an expansion of health insurance coverage, are needed. 

Third, the need factors related to unmet medical needs were regular exercise, pain, self-rated health status, and depression. The prevalence of unmet medical needs has been found to be higher among those with worse mobility or with chronic pain due to the resultant difficulties with mobility [43]. Past studies have also found unmet medical needs to be associated with worse self-rated health status [44] and depression [45,46] because of the costs related to depression [47,48]. 

When combining the results of this study and those of previous research, it appears that the factors that affect unmet medical need are older age, lower education level, a lack of economic activity, worse self-rated health status, and severe depression. Socially and economically vulnerable classes tend to require more health care and experience more health problems [15]. One way of addressing this problem is through cost sharing, which enables greater access of medical care by a particular demographic group. Therefore, policies should be established to reduce medical costs or enhance coverage of elderly and vulnerable populations, which may help to enhance equality in the use of medical care.

This research has several limitations. First, the results of this study cannot be easily generalized, as they are likely to be influenced by the cultural background and payment or compensation system of the country. Therefore, in future studies, it will be necessary to interpret the results in light of the country’s health care system. Second, among the factors that might influence unmet medical needs are distance to the medical facility and moral laxity due to private insurance [49]. However, these variables were not included in the health panel data, which means we could not control for them. Third, the KHPS is based on patient self-reports rather than on medical records. The accuracy of the survey data may be impaired by any of a number of sources of bias, such as recall bias. Also, operational definition was needed for variables with missing values is high(income) or rare (TM) through several expert meeting. Therefore, there may be problems with the accuracy of the data and this procedure might have introduced selection bias. Finally, because this study used a cross-sectional research design based on data from one year (2016), the causal relationships must be confirmed in future studies [50]. It might be possible to explore the trends in changes through a time-series analysis using accumulated longitudinal data in the future.

Despite these limitations, our study has significance for the following reasons. We analyzed the current status and multiple correlates of unmet medical needs using recent representative, data from the KHPS. Perhaps the greatest advantages of the study are that we examined the factors that affect unmet medical needs in detail and that we included factors such as non-covered treatments. In the future, policy studies are needed to evaluate and develop health policies on this topic, as current research in this area is insufficient. We believe that this study will be a high quality reference for countries with similar health systems to that of Korea—especially France, Germany, Japan, and Ireland—in which private insurance complements the public system’s cost-sharing obligations. 

## 5. Conclusions

The results of this study indicate that about 1 in 9 Korean citizens (11.6%) have unmet medical needs. In this study, the following three factors have been shown to be caused by unmet medical needs. First, regarding predisposing factors, with increased age, decreased levels of education, and the absence of a spouse, participants were more likely to experience unmet medical needs. Second, regarding need factors, participants experienced unmet medical needs when monthly household income was lower, medical benefits came from Medicaid, and non-covered treatments were more prevalent. Third, with regard to enabling factors, participants experienced unmet medical needs when they did not exercise, they experienced pain, their self-rated health status was worse, and they experienced severe depression. In conclusion, socially and economically vulnerable people experience more unmet medical needs than do others. Therefore, economic and public health approaches are necessary to reduce experiences with unmet medical needs; these include enhancement of health care coverage at the national level. 

## Figures and Tables

**Table 1:** Presence of unmet medical needs according to sociodemographic characteristics of participants.

Variables	Class	Total		Unmet Medical Needs				*p*
				No		Yes		
		*N*	%	*n*	%	*n*	%	
Total		11,378	(100.0)	10,058	(88.4)	1320	(11.6)	
Sex	Male	4816	(42.3)	4329	(43.0)	487	(36.9)	0.000
	Female	6562	(57.7)	5729	(57.0)	833	(63.1)	
Age (years)	20–29	826	(7.3)	774	(7.7)	52	(3.9)	0.000
	30–39	1087	(9.6)	989	(9.8)	98	(7.4)	
	40–49	2018	(17.7)	1805	(17.9)	213	(16.1)	
	50–59	2127	(18.7)	1889	(18.8)	238	(18.0)	
	60–69	2341	(20.6)	2073	(20.6)	268	(20.3)	
	70≦	2979	(26.2)	2528	(25.1)	451	(34.2)	
Education	Middle and high school graduate	3988	(34.0)	3385	(33.7)	603	(45.7)	0.000
	College or university graduate	3518	(30.9)	3134	(31.2)	384	(29.1)	
	Graduate university graduate	3872	(35.1)	3539	(35.2)	333	(25.2)	
Marital status	Married	8176	(71.9)	7323	(72.8)	853	(64.6)	0.000
	Unmarried	3202	(28.1)	2735	(27.2)	467	(35.4)	
Economic activity	Current worker	4267	(37.5)	3791	(37.7)	476	(36.1)	0.250
	Non-worker	7111	(62.5)	6267	(62.3)	844	(63.9)	
Monthly income (quintiles)	1Q (lowest)	4420	(38.8)	3792	(37.7)	628	(47.6)	0.000
	2Q	1709	(15.0)	1518	(15.1)	191	(14.5)	
	3Q	1720	(15.1)	1520	(15.1)	200	(15.2)	
	4Q	1772	(15.6)	1607	(16.0)	165	(12.5)	
	5Q (highest)	1757	(15.4)	1621	(16.1)	136	(10.3)	
Medical insurance type	NHI	7426	(65.3)	6571	(65.3)	855	(64.8)	0.000
	Medicaid	528	(4.6)	421	(4.2)	107	(8.1)	
	Others	3424	(30.1)	3066	(30.5)	358	(27.1)	
Private insurance	No	3140	(27.6)	2667	(26.5)	473	(35.8)	0.000
	Yes	8238	(72.4)	7391	(73.5)	847	(64.2)	
Non-covered treatment	No	3928	(34.5)	3462	(34.4)	466	(35.3)	0.526
	Yes	7450	(65.5)	6596	(65.6)	854	(64.7)	
Chronic disease	No	3280	(28.8)	3012	(29.9)	268	(20.3)	0.000
	Yes	8098	(71.2)	7046	(70.1)	1052	(79.7)	
Disability	No	10,510	(92.4)	9335	(92.8)	1175	(89.0)	0.000
	Yes	868	(7.6)	723	(7.2)	145	(11.0)	
Regular Exercise	No	1576	(78.7)	1257	(12.5)	319	(24.2)	0.000
	Yes	9802	(21.3)	8801	(87.5)	1001	(75.8)	
Pain	No	7832	(68.8)	7243	(72.0)	589	(44.6)	0.000
	Yes	3546	(31.2)	2815	(28.0)	731	(55.4)	
Self-rated health status	Good	4434	(39.0)	4137	(41.1)	297	(22.5)	0.000
	Moderate	4978	(43.8)	4399	(43.7)	579	(43.9)	
	Bad	1966	(17.3)	1522	(15.1)	444	(33.6)	
Depression	Light	9599	(84.4)	8705	(86.5)	894	(67.7)	0.000
	Moderate	1718	(15.1)	1312	(13.0)	406	(30.8)	
	Severe	61	(0.5)	41	(0.4)	20	(1.5)	
Type of medical care	TM & WM	2299	(20.2)	1985	(19.7)	314	(23.8)	0.002
	TM	80	(0.7)	68	(0.7)	12	(0.9)	
	WM	8761	(77.0)	7787	(77.4)	974	(73.8)	
	Other	238	(2.1)	218	(2.2)	20	(1.5)	

Notes: A chi-square test was used to determine differences in presence of unmet medical needs according to general characteristics. Abbreviations: NHI, National Health Insurance; TM, traditional Korean medicine; WM, Western medicine.

**Table 2:** Unmet medical needs according to sociodemographic characteristics, stratified by age.

Variables	Class	20–39 Years Old						40–59 Years Old						60 Years Old≦					
		Total	Unmet				*p*	Total	Unmet				*p*	Total	Unmet				*p*
			No		Yes				No		Yes				No		Yes		
		*N*	*N*	%	*n*	%		*N*	*n*	%	*n*	%		*N*	*n*	%	*n*	%	
Total		1913	1763	(92.2)	150	(7.8)		4145	3694	(89.1)	451	(10.9)		5320	4601	(86.5)	719	(13.5)	
Sex	Male	786	727	(41.2)	59	(39.3)	0.649	1787	1605	(43.4)	182	(40.4)	0.210	2243	1997	(43.4)	246	(34.2)	0.000
	Female	1127	1036	(58.8)	91	(60.7)		2358	2089	(56.6)	269	(59.6)		3077	2604	(56.6)	473	(65.8)	
Education	Graduate university graduate	16	15	(0.9)	1	(0.7)	0.267	434	372	(10.1)	62	(13.7)	0.031	3538	2998	(65.2)	540	(75.1)	0.000
	College or university graduate	364	328	(18.6)	36	(24.0)		1916	1705	(46.2)	211	(46.8)		1238	1101	(23.9)	137	(19.1)	
	Middle or high school graduate	1533	1420	(80.5)	113	(75.3)		1795	1617	(43.8)	178	(39.5)		544	502	(10.9)	42	(5.8)	
Marital status	Married	731	661	(37.5)	70	(46.7)	0.026	3645	3278	(88.7)	367	(81.4)	0.000	3800	3384	(73.5)	416	(57.9)	0.000
	Unmarried	1182	1102	(62.5)	80	(53.3)		500	416	(11.3)	84	(18.6)		1520	1217	(26.5)	303	(42.1)	
Economic activity	Current worker	438	391	(22.2)	47	(31.3)	0.010	1874	1664	(45.0)	210	(46.6)	0.541	1955	1736	(37.7)	219	(30.5)	0.000
	Non-worker	1475	1372	(77.8)	103	(68.7)		2271	2030	(55.0)	241	(53.4)		3365	2865	(62.3)	500	(69.5)	
Monthly income (quintiles)	1Q (lowest)	531	497	(28.2)	34	(22.7)	0.000	1256	1097	(29.7)	159	(35.3)	0.000	2633	2198	(47.8)	435	(60.5)	0.000
	2Q	203	184	(10.4)	19	(12.7)		448	395	(10.7)	53	(11.8)		1058	939	(20.4)	119	(16.6)	
	3Q	342	294	(16.7)	48	(32.0)		699	627	(17.0)	72	(16.0)		679	599	(13.0)	80	(11.1)	
	4Q	417	385	(21.8)	32	(21.3)		849	767	(20.8)	82	(18.2)		506	455	(9.9)	51	(7.1)	
	5Q (highest)	420	403	(22.9)	17	(11.3)		893	808	(21.9)	85	(18.8)		444	410	(8.9)	34	(4.7)	
Medical insurance type	NHI	795	725	(41.1)	70	(46.7)	0.384	2375	2119	(57.4)	256	(56.8)	0.006	4256	3727	(81.0)	529	(73.6)	0.000
	Medicaid	21	19	(1.1)	2	(1.3)		109	87	(2.4)	22	(4.9)		398	315	(6.8)	83	(11.5)	
	Others	1097	1019	(57.8)	78	(52.0)		1661	1488	(40.3)	173	(38.4)		666	559	(12.1)	107	(14.9)	
Private insurance	Yes	244	219	(12.4)	25	(16.7)	0.135	451	386	(10.4)	65	(14.4)	0.011	2445	2062	(44.8)	383	(53.3)	0.000
	No	1669	1544	(87.6)	125	(83.3)		3694	3308	(89.6)	386	(85.6)		2875	2539	(55.2)	336	(46.7)	
Non-covered treatment	No	912	841	(47.7)	71	(47.3)	0.931	1612	1423	(38.8)	180	(39.9)	0.638	1404	1189	(25.8)	215	(29.9)	0.022
	Yes	1001	922	(52.3)	79	(52.7)		2533	2262	(61.2)	271	(60.1)		3916	3412	(74.2)	504	(70.1)	
Chronic disease	No	1322	1222	(69.3)	100	(66.7)	0.501	1630	1492	(40.4)	138	(30.6)	0.000	328	298	(6.5)	30	(4.2)	0.017
	Yes	591	541	(30.7)	50	(33.3)		2515	2202	(59.6)	313	(69.4)		4992	4303	(93.5)	689	(95.8)	
Disability	Yes	1878	1731	(98.2)	147	(98.0)	0.871	3978	3561	(96.4)	417	(92.5)	0.000	4654	4043	(87.9)	611	(85.0)	0.029
	No	35	32	(1.8)	3	(2.0)		167	133	(3.6)	34	(7.5)		666	558	(12.1)	108	(15.0)	
Regular exercise	Yes	18	15	(0.9)	3	(2.0)	0.162	138	112	(3.0)	26	(5.8)	0.002	1420	1130	(24.6)	290	(40.3)	0.000
	No	1895	1748	(99.1)	147	(98.0)		4007	3582	(97.0)	425	(94.2)		3900	3471	(75.4)	429	(59.7)	
Pain	No	1758	1635	(92.7)	123	(82.0)	0.000	3350	3083	(83.5)	267	(59.2)	0.000	2724	2525	(54.9)	199	(27.7)	0.000
	Yes	155	128	(7.3)	27	(18.0)		795	611	(16.5)	184	(40.8)		2596	2076	(45.1)	520	(72.3)	
Self-rated health status	Good	1163	1097	(62.2)	66	(44.0)	0.000	1837	1706	(46.2)	131	(29.0)	0.000	1434	1334	(29.0)	100	(13.9)	0.000
	Moderate	633	571	(32.4)	62	(41.3)		1930	1697	(45.9)	233	(51.7)		2415	2131	(46.3)	284	(39.5)	
	Bad	117	95	(5.4)	22	(14.7)		378	291	(7.9)	87	(19.3)		1471	1136	(24.7)	335	(46.6)	
Depression	Light	1760	1636	(92.8)	124	(82.7)	0.000	3656	3305	(89.5)	351	(77.8)	0.000	4183	3764	(81.8)	419	(58.3)	0.000
	Moderate	146	121	(6.9)	25	(16.7)		476	379	(10.3)	97	(21.5)		1096	812	(17.6)	284	(39.5)	
	Severe	7	6	(0.3)	1	(0.7)		13	10	(0.3)	3	(0.7)		41	25	(0.5)	16	(2.2)	
Type of medical care	TM & WM	228	206	(11.7)	22	(14.7)	0.434	728	621	(16.8)	107	(23.7)	0.001	1343	1158	(25.2)	185	(25.7)	0.853
	TM	22	19	(1.1)	3	(2.0)		45	38	(1.0)	7	(1.6)		13	11	(0.2)	2	(0.3)	
	WM	1590	1469	(83.3)	121	(80.7)		3266	2935	(79.5)	331	(73.4)		3905	3383	(73.5)	522	(72.6)	
	EM	73	69	(3.9)	4	(2.7)		106	100	(2.7)	6	(1.3)		59	49	(1.1)	10	(1.4)	

Notes: A chi-square test was used to determine differences in presence of unmet medical needs according to general characteristics and age. Abbreviations: NHI, National Health Insurance; TM, Korean traditional medicine; WM, Western medicine; EM, Etc. medicine.

**Table 3:** Regression analysis of the factors related to unmet medical needs.

Variables		Odds Ratio (95% Confidence Interval)											
		Model 1				Model 2				Model 3			
		OR			*p*-Value	OR			*p*-Value	OR			*p*-Value
Gender	Male	1.00				1.00				1.00			
	Female	1.13	1.00	1.28	0.059	1.18	1.04	1.34	0.011	1.09	0.96	1.25	0.185
Age (years)	20–29	1.00				1.00				1.00			
	30–39	1.93	1.35	2.75	0.000	1.90	1.32	2.71	0.000	1.72	1.19	2.47	0.004
	40–49	2.48	1.77	3.47	0.000	2.40	1.32	3.36	0.000	1.99	1.41	2.82	0.000
	50–59	2.43	1.72	3.42	0.000	2.42	1.32	3.42	0.000	1.76	1.23	2.52	0.002
	60–69	2.14	1.50	3.05	0.000	2.17	1.32	3.10	0.000	1.53	1.05	2.23	0.026
	70≦	2.51	1.78	3.56	0.000	2.28	1.32	3.28	0.000	1.43	0.97	2.10	0.067
Education	Graduate university graduate	1.00				1.00				1.00			
	College or universitygraduate	1.21	1.02	1.43	0.027	1.17	0.98	1.38	0.076	1.09	0.92	1.30	0.334
	Middle and high school graduate	1.51	1.24	1.85	0.000	1.39	1.13	1.71	0.002	1.09	0.88	1.35	0.424
Marital status	Married	1.00				1.00				1.00			
	Unmarried	1.63	1.42	1.86	0.000	1.52	1.33	1.75	0.000	1.42	1.23	1.63	0.000
Economic	Current worker					1.00				1.00			
activity	Non-worker					1.07	0.92	1.24	0.382	1.24	1.06	1.46	0.007
Monthly income (quintiles)	5Q (highest)					1.00				1.00			
	4Q					1.19	0.93	1.51	0.162	1.19	0.93	1.51	0.171
	3Q					1.42	1.12	1.79	0.003	1.43	1.13	1.81	0.003
	2Q					1.18	0.93	1.50	0.172	1.11	0.87	1.42	0.408
	1Q (lowest)					1.49	1.21	1.84	0.000	1.41	1.14	1.75	0.001
Medical insurance type	NHI					1.00				1.00			
	Medicaid					1.43	1.13	1.82	0.003	1.48	1.22	1.78	0.000
	Others					1.12	0.94	1.34	0.206	1.11	0.87	1.43	0.404
Private insurance	Yes					1.00				1.00			
	No					1.17	1.01	1.36	0.033	1.06	0.91	1.23	0.488
Non-covered treatment	No					1.00				1.00			
	Yes					1.11	0.97	1.26	0.120	1.24	1.08	1.42	0.002
Chronic disease	No									1.00			
	Yes									1.09	0.91	1.30	0.342
Disability	No									1.00			
	Yes									0.99	0.80	1.22	0.932
Regular Exercise	Yes									1.00			
	No									1.23	1.02	1.48	0.027
Pain	No									1.00			
	Yes									2.29	1.97	2.66	0.000
Self-rated Health Status	Good									1.00			
	Moderate									1.46	1.25	1.71	0.000
	Bad									2.29	1.89	2.79	0.000
Depression	Light									1.00			
	Moderate									1.79	1.54	2.07	0.000
	Severe									2.46	1.39	4.35	0.002
AUC *		0.600				0.612				0.700			

Notes: A logistic regression analysis with a complex sampling design was performed after adjusting for covariates. OR, odds ratio; 95% CI, 95% confidence interval. Model 1 was adjusted for sex, age, and marital status. Model 2 was adjusted for the Model 1 variables + economic activity, monthly income, medical insurance type, private insurance and non-covered treatment. Model 3 was adjusted for the Model 2 variables + chronic disease, disability, regular exercise, pain, self-rated health status, and depression. * The AUC (area under the receiver operating characteristic curve) indicates the discrimination ability of the prediction model. Abbreviations: NHI, National Health Insurance; OR, Odds Ratio; 95% CI, 95% confidence interval.

**Table 4:** Regression analysis of the factors that affect unmet medical needs according to age group.

Variables		19–39 Years Old				40–59 Years Old				≥60 Years Old			
		OR			*p*	OR			*p*	OR			*p*
Gender	Male	1.00				1.00				1.00			
	Female	0.98	0.68	1.41	0.921	1.14	0.92	1.41	0.248	1.00	0.82	1.21	0.960
Education	Graduate university graduate	1.00				1.00				1.00			
	College or university graduate	0.56	0.07	4.68	0.590	1.00	0.70	1.43	0.991	1.31	0.91	1.88	0.142
	Middle and high school graduate	1.19	0.79	1.81	0.405	1.01	0.80	1.26	0.956	1.30	0.89	1.90	0.170
Marital status	Married	1.00				1.00				1.00			
	Unmarried	0.82	0.52	1.29	0.395	1.41	1.06	1.87	0.018	1.59	1.31	1.92	0.000
Economic activity	Current worker	1.00				1.00				1.00			
	Non-worker	1.97	1.15	3.35	0.013	1.37	0.99	1.90	0.057	1.12	0.91	1.37	0.283
Monthly income (quintiles)	5Q (highest)	1.00				1.00				1.00			
	4Q	1.91	1.03	3.54	0.039	1.05	0.75	1.45	0.789	1.14	0.71	1.82	0.599
	3Q	3.83	2.12	6.91	0.000	1.05	0.74	1.48	0.800	1.30	0.84	2.01	0.246
	2Q	2.32	1.14	4.71	0.020	1.03	0.70	1.51	0.897	1.07	0.70	1.62	0.767
	1Q (lowest)	1.54	0.84	2.85	0.165	1.20	0.89	1.61	0.228	1.51	1.02	2.23	0.040
Medical insurance type	NHI	1.00				1.00				1.00			
	Medicaid	1.13	0.23	5.43	0.882	1.55	1.09	2.21	0.015	1.84	1.41	2.42	0.000
	Others	1.30	0.77	2.20	0.335	0.88	0.49	1.57	0.662	1.15	0.87	1.53	0.330
Private insurance	Yes	1.00				1.00				1.00			
	No	1.36	0.84	2.21	0.214	1.10	0.80	1.51	0.548	0.97	0.80	1.17	0.734
Non-covered treatment	No	1.00				1.00				1.00			
	Yes	1.22	0.83	1.80	0.312	1.19	0.95	1.50	0.123	**1.31**	**1.08**	**1.58**	**0.006**
Chronic disease	No	1.00				1.00				1.00			
	Yes	0.92	0.63	1.35	0.656	1.22	0.96	1.55	0.098	1.01	0.66	1.53	0.972
Disability	No	1.00				1.00				1.00			
	Yes	0.54	0.14	2.02	0.360	1.62	1.04	2.55	0.035	0.89	0.70	1.14	0.361
Regular exercise	Yes	1.00				1.00				1.00			
	No	0.90	0.22	3.79	0.887	1.68	1.02	2.77	0.041	1.21	0.98	1.49	0.074
Pain	No	1.00				1.00				1.00			
	Yes	1.43	0.80	2.53	0.226	2.82	2.22	3.59	0.000	2.14	1.73	2.64	0.000
Self-rated health status	Good	1.00				1.00				1.00			
	Moderate	1.52	1.04	2.23	0.033	1.46	1.15	1.85	0.002	1.47	1.15	1.90	0.002
	Bad	2.43	1.32	4.48	0.004	2.23	1.57	3.16	0.000	2.34	1.77	3.08	0.000
Depression	Light	1.00				1.00				1.00			
	Moderate	2.26	1.31	3.91	0.003	1.43	1.08	1.89	0.013	1.97	1.63	2.39	0.000
	Severe	1.62	0.18	14.73	0.667	1.22	0.31	4.86	0.779	3.68	1.87	7.21	0.000

Notes: A logistic regression analysis with a complex sampling design was performed, adjusting for all covariates. Abbreviations: NHI, National Health Insurance; OR, odds ratio; 95% CI, 95% confidence interval.

**Table 5:** Association between non-covered treatment and unmet medical needs according to sex, age, and medical type.

Class	Category			Model 1				Model 2				Model 3			
				OR	95% CI		*p*	OR	95% CI		*p*	OR	95% CI		*p*
Sex	Male	Non-covered treatment	Yes	1.37	1.13	1.66	0.002	1.30	1.06	1.59	0.011	1.47	1.19	1.82	0.000
			No	1.00				1.00				1.00			
	Female	Non-covered treatment	Yes	1.00	0.85	1.18	0.969	0.99	0.83	1.18	0.915	1.11	0.93	1.33	0.244
			No	1.00				1.00				1.00			
Age	19–39	Non-covered treatment	Yes	1.05	0.75	1.48	0.768	1.15	0.79	1.67	0.481	1.22	0.83	1.80	0.312
			No	1.00				1.00				1.00			
	40–59	Non-covered treatment	Yes	1.07	0.88	1.32	0.499	1.08	0.87	1.34	0.512	1.19	0.95	1.50	0.123
			No	1.00				1.00				1.00			
	60≦	Non-covered treatment	Yes	1.23	1.03	1.47	0.020	1.15	0.96	1.38	0.041	1.31	1.08	1.58	0.006
			No	1.00				1.00				1.00			
Type of medical	TM	Non-covered treatment	Yes	0.91	0.68	1.22	0.554	0.83	0.61	1.14	0.261	0.9	0.65	1.23	0.517
			No	1.00				1.00				1.00			
	WM	Non-covered treatment	Yes	1.22	1.06	1.40	0.004	1.19	1.02	1.37	0.019	1.33	1.15	1.55	0.000
			No	1.00				1.00				1.00			

Notes: Logistic regression analysis with complex sampling design was performed by adjusting for covariates. Odds ratios indicates odds ratio; 95% CI, 95% confidence interval. Model 1 was adjusted for sex, age, and marital status. Model 2 was adjusted by Model 1 variables + economic activity, monthly income, medical insurance type, private insurance, and non-covered treatment. Model 3 was adjusted by Model 2 variables + chronic disease, disability, regular exercise, pain, self-rated health status, and depression. Abbreviations: TM, Korean traditional medicine; WM, Western medicine; OR, odds ratio; 95% CI, 95% confidence interval.

## Appendix A

**Table A1:** Medical costs of participants with unmet needs and those with met needs according to type of medical care.

Category	Met Needs		Unmet Needs	
	Mean	SD	Mean	SD
TM_Outpatient visits	2.27	9.303	3.22	11.961
TM_Total cost *	35.90	167.56	51.84	200.79
TM_INSUP cost ^†^	26.53	123.22	41.04	170.70
TM_SLF cost ^‡^	6.08	29.25	7.50	27.91
TM_Uncovered cost ^§^	3.31	57.68	3.30	34.91
WM_Outpatient visits	17.31	23.022	19.87	26.565
WM_Total *	576.28	7848.72	529.80	1146.17
WM_INSUP cost ^†^	358.00	7816.19	302.50	936.14
WM_SLF ^‡^	106.97	182.09	116.32	190.84
WM_Uncovered cost ^§^	111.39	329.53	111.27	250.60

* Total cost: Sum of INSUP cost and SLF cost paid to the medical care institution. What is determined to be eligible for reimbursement by the Health Insurance Review and Assessment Service (HIRA) out of the total treatment amount is indicated in the submitted insurance claim statement. We converted costs into 2019 U.S. dollars (1 USD = 1142.30 KRW as of April 10, 2019). ^†^ INSUP cost: Cost reimbursed by the Korean National Health Insurance Service as the insurer. ^‡^ SLF cost: Self-payment amount paid by the beneficiary. ^§^ Non-covered cost: Costs not guaranteed by National Health Insurance and of which the patient must pay 100%. Abbreviations: TM, traditional Korean medicine; WM, Western medicine.

**Table A2:** Factors that affect general perceived unmet needs.

Reason for Unmet Needs	*n*	(%)
Financial reasons (burden of treatment costs)	380	(28.8)
Medical facility is too far away	40	(3.0)
Difficulty with mobility and difficulty visiting due to health reasons	98	(7.4)
There is no one to care for the children	22	(1.7)
Symptoms are minor	361	(27.3)
Do not know where to go (lack of information)	9	(0.7)
Lack of time to visit	357	(27.0)
Appointments cannot be made in a timely manner	1	(0.1)
There is no attending physician	2	(0.2)
Other	50	(3.8)
Total	1320	(100.0)

**Table A3:** Factors affecting unmet needs.

Variables		Odds Ratio (95% Confidence Interval)							
		Model 1: Unadjusted OR				Model 2: Adjusted OR			
		OR			*p*	OR			*p*
Gender	Male	1.00				1.00			
	Female	1.29	1.15	1.46	0.000	1.09	0.96	1.25	0.185
Age (years)	20–29	1.00				1.00			
	30–39	1.47	1.04	2.09	0.029	1.72	1.19	2.47	0.004
	40–49	1.76	1.28	2.41	0.000	1.99	1.41	2.82	0.000
	50–59	1.88	1.37	2.56	0.000	1.76	1.23	2.52	0.002
	60–69	1.92	1.41	2.62	0.000	1.53	1.05	2.23	0.026
	70≦	2.66	1.97	3.58	0.000	1.43	0.97	2.10	0.067
Education	Graduate university graduate	1.00				1.00			
	College or university graduate	1.30	1.12	1.52	0.001	1.09	0.92	1.30	0.334
	Middle and high school graduate	1.89	1.64	2.18	0.000	1.09	0.88	1.35	0.424
Marital status	Married	1.00				1.00			
	Unmarried	1.47	1.30	1.65	0.000	1.42	1.23	1.63	0.000
Economic activity	Current worker	1.00				1.00			
	Non-worker	1.07	0.95	1.21	0.250	1.24	1.06	1.46	0.007
Income (quintiles)	5Q (highest)	1.00				1.00			
	4Q	1.22	0.97	1.55	0.095	1.19	0.93	1.51	0.171
	3Q	1.57	1.25	1.97	0.000	1.43	1.13	1.81	0.003
	2Q	1.50	1.19	1.89	0.001	1.11	0.87	1.42	0.408
	1Q (lowest)	1.97	1.63	2.40	0.000	1.41	1.14	1.75	0.001
Medical insurance type	NHI	1.00				1.00			
	Medicaid	1.95	1.56	2.44	0.000	1.48	1.22	1.78	0.000
	Others	0.90	0.79	1.02	0.104	1.11	0.87	1.43	0.404
Private insurance	Yes	1.00				1.00			
	No	1.55	1.37	1.75	0.000	1.06	0.91	1.23	0.488
Non-covered treatment	No	1.00				1.00			
	Yes	1.17	0.97	1.41	0.100	1.24	1.08	1.42	0.002
Chronic Disease	No	1.00				1.00			
	Yes	1.68	1.46	1.93	0.000	1.09	0.91	1.30	0.342
Disability	Yes	1.00				1.00			
	No	1.59	1.32	1.92	0.000	0.99	0.80	1.22	0.932
Regular exercise	Yes	1.00				1.00			
	No	1.18	1.02	1.37	0.025	1.23	1.02	1.48	0.027
Pain	No	1.00				1.00			
	Yes	3.19	2.84	3.59	0.000	2.29	1.97	2.66	0.000
Self-rated health status	Good	1.00				1.00			
	Moderate	1.83	1.58	2.12	0.000	1.46	1.25	1.71	0.000
	Bad	4.06	3.47	4.76	0.000	2.29	1.89	2.79	0.000
Depression	Light	1.00				1.00			
	Moderate	3.01	2.64	3.43	0.000	1.79	1.54	2.07	0.000
	Severe	4.75	2.77	8.14	0.000	2.46	1.39	4.35	0.002
AUC *						0.700			

Notes: A logistic regression analysis with complex sampling design was performed after adjusting for all covariates. Abbreviations: NHI, National Health Insurance; OR, odds ratio; 95% CI, 95% confidence intervals.
